# Omalizumab Treated Urticaria Patients Display T Cell and Thrombocyte‐Associated Gene Regulation

**DOI:** 10.1002/iid3.70132

**Published:** 2025-02-11

**Authors:** Anna Smola, Heike C. Hawerkamp, Péter Oláh, Andreas Kislat, Nicole Duschner, Bernhard Homey, Stephan Meller

**Affiliations:** ^1^ Department of Dermatology Medical Faculty Heinrich‐Heine‐University Duesseldorf Germany; ^2^ Department of Dermatology, Venereology and Oncodermatology University of Pécs Pécs Hungary

## Abstract

**Background:**

Chronic spontaneous urticaria (CSU) is a debilitating inflammatory skin disease with a prevalence of approximately 1% of the population. It is characterized by recurrent itchy wheals and/or angioedema for more than 6 weeks without known triggers leading to a high quality of life impairment. The pathogenesis of CSU remains not fully understood.

**Objective:**

This study aimed to explore the pathomechanism of CSU beyond mast cells and IgE‐dependent histamine release and to identify possible biomarkers for the disease and its treatment.

**Methods:**

We investigated a patient cohort in the first month of omalizumab treatment regarding the IgE levels and changes in gene and miRNA expression in peripheral blood. The cohort was divided into responders and nonresponders (depending on the score of the urticaria control test) and compared to a group of healthy controls.

**Results:**

Our messenger RNA and microRNA microarray analyses revealed the greatest changes in expression levels on Day 2 after the first omalizumab dose.

**Conclusion:**

We identified several genes and miRNAs of interest, most of which have not been described to be linked to CSU so far, underlining, for example, to T cell involvement or even suggesting platelet involvement.

## Graphical abstract

Chronic spontaneous urticaria is a debilitating inflammatory skin leading to a high quality of life impairment. We investigated a omalizumab treated cohort in regard to changes of gene and miRNA expression in peripheral blood. Our results point to T cell involvement and suggest platelet involvement. Created in BioRender. Hawerkamp, H. (2025). https://BioRender.com/v03d595

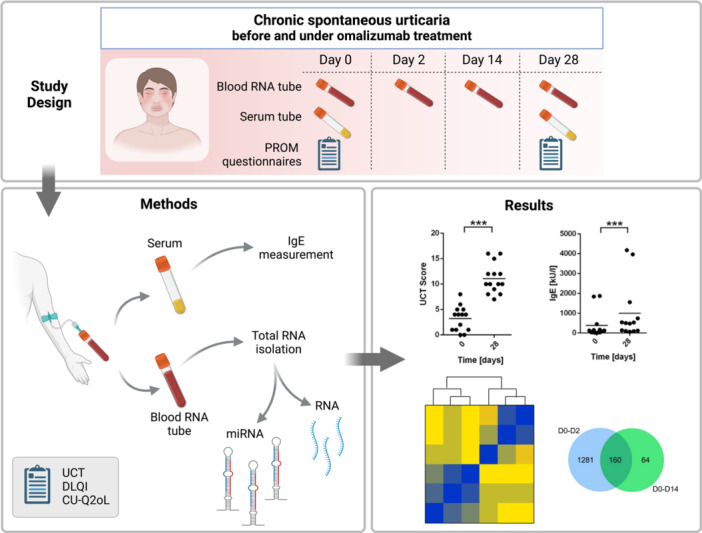

## 1 Introduction

Chronic spontaneous urticaria (CSU) is a subtype of urticaria, an inflammatory skin disease which has a worldwide prevalence of approximately 1% [1]. CSU is characterized by recurrent itchy wheals or angioedema or both for longer than 6 weeks with no specific triggers [2]. CSU is associated with autoimmune diseases with studies showing that up to one‐third of CSU patients have an autoimmune comorbidity—most common being thyroid disease [3, 4]. Patients with CSU suffer from severe quality of life impairment due to the unpredictability of the symptoms leading to a burden of healthcare, society, and economy [5, 6].

In recent years, patient‐reported outcome measures (PROMs) in the form of questionnaires, simple yes/no questions, or rating scales have become an essential tool in clinical trials and clinical care [7]. In dermatology, the most used PROM instrument is the dermatology life quality index (DLQI) which has been validated for urticaria patients [8]. Other tools are the urticaria control test (UCT) and the chronic urticaria quality of life questionnaire (CU‐Q2oL) [9, 10]. On a cellular level, CSU is considered a mast cell‐driven disease [2, 11]. Mast cells (MC) can be activated by Immunoglobin E (IgE) through IgE cross‐linking and binding to FcεRI, the high‐affinity IgE receptor found on the surface of MCs [12, 13]. Studies have shown that many CSU patients have elevated serum levels of total and free IgE [14, 15]. MCs can additionally be activated by various other, non‐IgE‐dependent mechanisms via several different receptors, for example, IL‐4 receptor α (IL‐4Rα) via IL‐13 but also histamine‐4‐receptor (H4R) via histamine [11, 16, 17]. Even though MCs are thought to be the predominant key effector cells, various other cells such as basophils and eosinophils are involved in urticaria as well [13]. Besides MCs, T cells also have been found to play a role in CSU. The infiltrating cells in skin biopsies taken from CSU patients are mostly T cells, with T_H_2 cells being the most prominent together with some T_H_1 and T_H_17 cells [18].

Although recent research has provided new insights on MC activation and other cells involved in the disease pathogenesis, the etiology remains not fully understood and is lacking specific biomarkers to assess treatment possibilities [2]. Some of this biomarker research focuses on molecular interactions via creation of molecular networks, for example, microRNA (miRNA) and their interaction with messenger RNA (mRNA) [19, 20]. MiRNAs are composed of 22−24 nucleotides and belong to the group of noncoding RNAs. MiRNAs are thought to have a regulatory effect on both the adaptive and the innate immune systems [21]. Modified miRNA expressions were shown for different conditions, including autoimmune diseases and allergic diseases [19, 20, 22, 23]. As miRNAs can be found in all body fluids and remain relatively stable, they are an optimal target in the search for biomarkers [24].

The current systemic treatment of CSU is based on antihistamines. However, many patients (up to 45%−50%) do not sufficiently respond to an up‐dosed antihistamine treatment [25]. In this case, the use of omalizumab, a monoclonal anti‐IgE‐antibody approved for CSU since 2014, is recommended [26].

Omalizumab binds to free IgE at its Cε3 domain which in turn leads to FcεRI downregulation and reduced MC and basophil activation [27]. The binding to free IgE also reduces IgE interaction with the low‐affinity IgE receptor (FcεRII) commonly known as CD23, found on B cells or eosinophils [28, 29]. This study aimed to explore CSU pathomechanism beyond MCs and IgE‐dependent histamine release. We investigated a patient cohort in the first month of omalizumab treatment by analyzing (1) PROMs, (2) IgE levels, (3) gene expression, and (4) miRNA expression in peripheral blood. We found most of our patient cohort responsive to omalizumab treatment based on PROM results and IgE levels. Furthermore, numerous genes and miRNAs were differentially regulated especially at Day 2 after treatment initiation.

## 2 Materials and Methods

### 2.1 Samples From Healthy Controls and Urticaria Patients

This study was approved by the local ethics committee of the medical faculty of the Heinrich‐Heine‐University Duesseldorf (No: 4676), and all experiments were performed following the guidelines of the Declaration of Helsinki. All patients provided written informed consent prior to study participation. Seventeen patients with antihistamine‐resistant CSU (diagnosis based on the criteria of the EAACI/GA²LEN/EDF/WAO guideline [2]) were recruited at the Department of Dermatology of the University Hospital Duesseldorf before starting omalizumab 300 mg every 4 weeks. Patients were advised to continue background treatment with antihistamines as omalizumab is an add‐on therapy to fourfold antihistamine‐intake daily. Peripheral blood was obtained prior to the first omalizumab dose (Day 0; D0), 2 days (D2), and 14 days (D14) after treatment initiation, and prior to the second omalizumab dose at Day 28 (D28). As omalizumab is an add‐on therapy, patients could take second‐generation antihistamines during the study period.

Peripheral blood was also collected from eight healthy individuals aged > 18 years as controls. They had no known history of urticaria or atopic diseases.

For blood collection, a Vacutainer blood collection tube (Becton, Dickinson and Company; Plymouth, United Kingdom) was used to obtain serum, and a PAXgene Blood RNA was used for the subsequent analysis of total RNA.

### 2.2 PROM Questionnaires

Disease control and quality of life impairment were documented by patients filling in the German versions of the UCT, CU‐Q2oL, and DLQI at D0 and D28. Responders were defined by a recorded increase of a minimum of 3 points of the UCT. Patients with no documented increase or even a documented decrease in the UCT score at D28 were considered nonresponders.

### 2.3 Measurement of Total Serum IgE

Blood sera from D0 and D28 were used for the measurement of total serum IgE levels by immunoassay (ImmunoCap; Phadia/Thermo Fisher Scientific, Waltham, USA) according to the manufacturer's protocol.

### 2.4 RNA Isolation

Total RNA (including mRNA and miRNA) was isolated using the PAXgene Blood miRNA Kit (PreAnalytiX; Hombrechtikon, Switzerland) according to the manufacturer's instructions from the peripheral blood collected into PAXgene Blood RNA tubes.

### 2.5 Microarray Analysis

Microarray analysis of mRNA samples was performed using the Affymetrix Human PrimeView 2.0 chip (Thermo Fisher Scientific, Waltham, USA). The miRNA samples were screened on Affymetrix GeneChip miRNA 4.0 Array (Thermo Fisher Scientific). RNA or miRNA quality was assessed prior to hybridization at the Biomedical Research Centre (Biomedizinisches Forschungszentrum [BMFZ]) at the Heinrich‐Heine‐University, Düsseldorf.

### 2.6 Statistical Analysis

#### 2.6.1 Analysis of Microarray Data

All arrays were submitted to RMA normalization and underwent ArrayQualityMetrics quality control. The analysis for differentially expressed genes (DEG) was conducted using the limma R package. Heatmaps, networks, and Venn diagrams were generated in R v3.5.

#### 2.6.2 Analysis of PROMs and Total IgE Levels

The patient questionnaires as well as total serum IgE levels were analyzed using GraphPad Prism Version 5.03 (GraphPad Software; San Diego, USA). For PROM results and total serum IgE levels, the Wilcoxon matched‐pairs signed rank test was used.

## 3 Results

### 3.1 Patient Characteristics

Seventeen patients in total were enrolled in this study: 11 female and six male patients with a range of 19−69 years and a disease duration range of 3−300 months (Table 1). Eleven patients had wheals and angioedema, six patients—wheals only. In all patients, disease control had not been achieved by fourfold standard dose treatment with antihistamines, whereas four patients additionally had montelukast. All patients were put on short or long‐term systemic corticosteroid treatment at some point during the past. Any patients with current corticosteroid treatment were advised to discontinue corticosteroids 4 weeks before starting omalizumab. Three patients suffered from thyroid disease and two from NSAID intolerance. Four patients had different type I hypersensitivities. Three patients had received treatment for *H. pylori* as a diagnostic workup in CSU, however, with no benefit for the CSU itself. Six patients had no reported concomitant diseases, medications, or allergies.

**Table 1:** List of anamnestic characteristics of the patient cohort.

Patient characteristics	
Sex ratio [male to female]	6:11 (36:64%)
Age [range in years]	19−69
Duration of CSU [range in months]	3−300
Wheals and AE [# of patients]	11 (65%)
Wheals only [# of patients]	6 (35%)
On treatment with fourfold standard dose of AH [# of patients]	17 (100%)
Any previous treatment with systemic CS [# of patients]	17 (100%)
On treatment with montelukast	4 (24%)
Thyroid disease	3 (18%)
NSAID intolerance [# of patients]	2 (12%)
Type I hypersensitivities/intolerances [# of patients]	4 (24%)
Treatment for *H. pylori* infection [# of patients]	3 (18%)
No known concomitant diseases/medications/allergies [# of patients]	6 (35%)

At D28, seven patients discontinued background medication with antihistamines, whereas 10 patients continued background treatment with antihistamines (dosage varied, mediation taken as needed). All responders chosen for the DNA and miRNA analysis discontinued antihistamines.

### 3.2 PROM Scores and Total Serum IgE Levels Underline Effectiveness of Omalizumab

**Figure 1:**
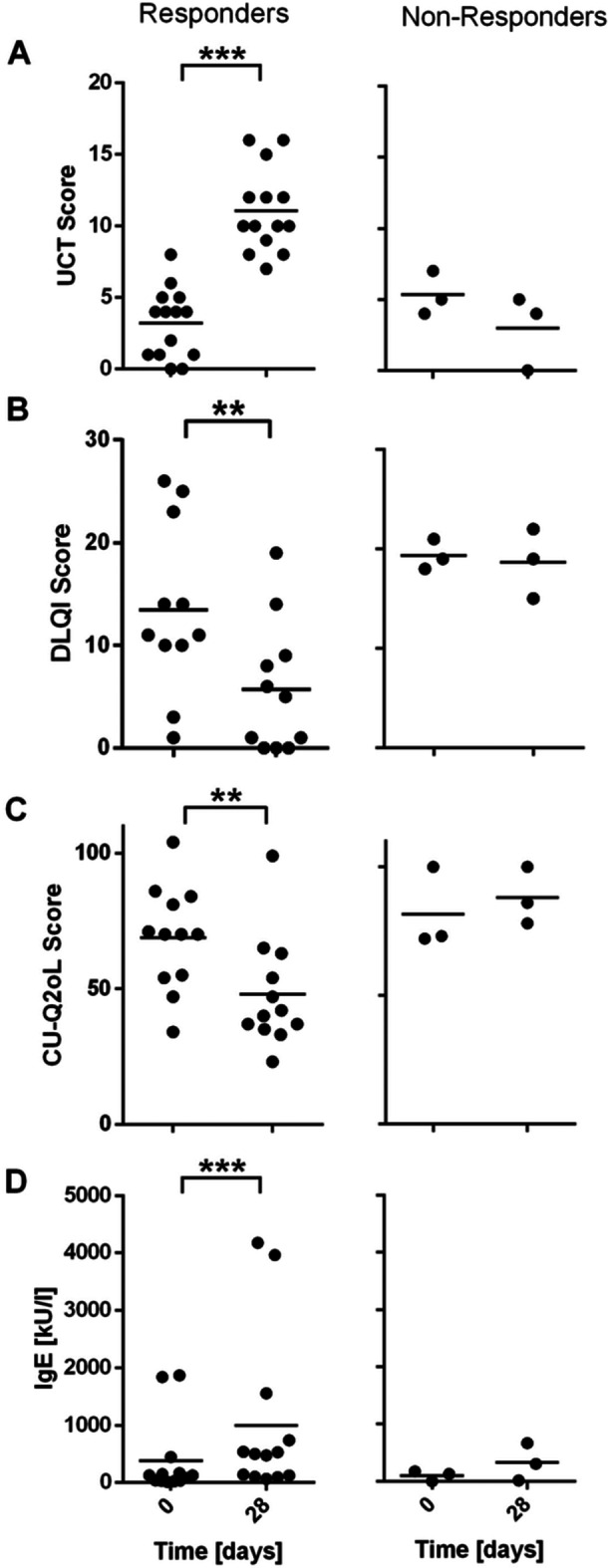
Increase of total UCT score and total IgE levels; reduction of DLQI and CU‐Q2oL scores at D28 compared to D0. Disease control and quality of life impairment in CSU cohort at D0 and D28 for UCT (A, *n* = 17), DLQI (B, *n* = 14), and CU‐Q2oL (C, *n* = 15). Patients with no change or decrease in UCT value at D28 are considered nonresponders. Total serum IgE levels in kU/L at baseline (D0) compared to D28 (D, *n* = 16). The dots represent individual patients with a horizontal line indicating mean value. Statistical analysis was performed using the Wilcoxon matched‐pairs signed‐rank test (***p ≤ 0.001, **p ≤ 0.01, *p ≤ 0.05). CSU = chronic spontaneous urticaria, CU‐Q2oL = chronic urticaria quality of life questionnaire, DLQI = dermatology life quality index, IgE = immunoglobulin E, UCT = urticaria control test.

PROM scores (UCT, DLQI, and CU‐Q2oL) compared D0 and D28 showed a significant amelioration of symptoms in our cohort (UCT *p *< 0.001, DLQI *p *< 0.01, CU‐Q2oL *p *< 0.05, Figure 1A−C). Three nonresponders were defined by the same score or decrease in UCT value at D28; correspondingly, these three patients also showed no decrease in DLQI and CU‐Q2oL scores. Thus, these three patients were determined as nonresponders for all further analyses.

Total serum IgE levels were measured for 16 patients at D0 and D28 showing a significant increase after 4 weeks of omalizumab treatment (*p *< 0.0001, Figure 1D). At baseline, the range of total serum IgE levels was 3.97−1867 kU/L. All but one patient (a responder) showed an increase in total serum IgE levels at D28 [range: 16.5−4177, mean: 875.15, and median: 490 kU/L]. Notably, three of the responders were also in the group of total serum IgE levels between 10 and 40 kU/L.

In the described cohort, no correlation between the total IgE levels and age or disease severity (compared to UCT, CU‐Q2oL, and DLQI scores at baseline) was observed (data not shown).

### 3.3 DNA Microarray Depicts a Complex Regulation of Gene Expression

**Figure 2:**
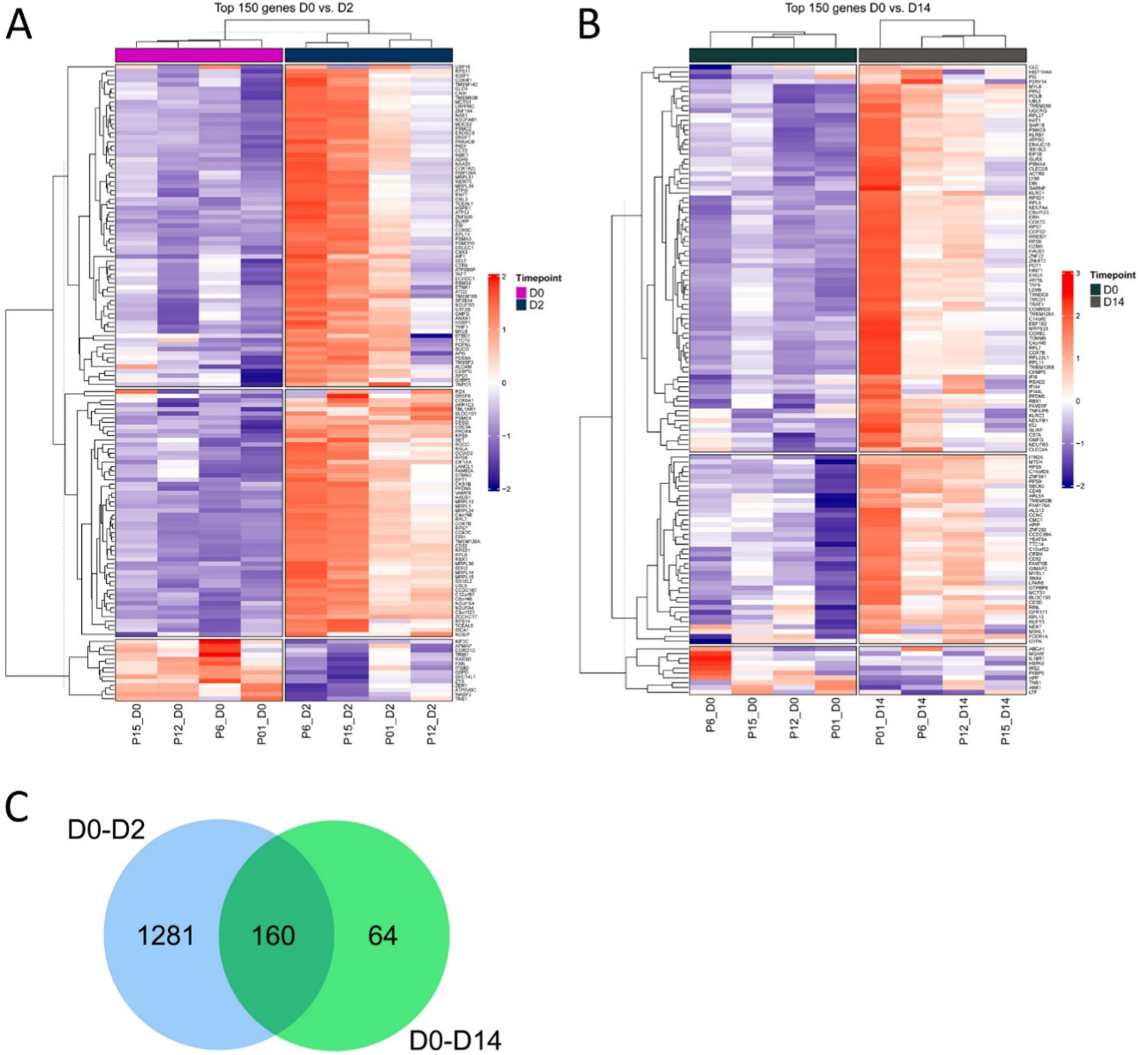
Differential gene regulation at D2 and D14 compared to D0 under omalizumab treatment. Heat map depicting top regulated DEGs at D2 (A), and D14 (B) compared to D0 based on DNA microarray analysis. Red color indicates gene upregulation, purple color indicates gene downregulation, and white color indicates regulation in gene expression. The Venn diagram (C) displays the overlapping DEGs at D0 compared to D2 (left side), and D14 (right side). The overlap of both sides shows that 160 DEGs are regulated in both analyzed data sets (D0 to D2, and D0 to D14). CSU = chronic spontaneous urticaria, D (0, 2, 14) = day (0, 2, 14), DEG = differentially expressed genes, P = patient.

Four responders were included in the DNA microarray analyses looking into changes between D0 to D2, and D0 to D14 under omalizumab treatment. Both analyses showed mostly upregulation in DEGs, especially for the comparison of D0 to D2. For visualization of the top 150 regulated genes, heatmaps were used to compare D0 to D2 (Figure 2A), and D0 to D14 (Figure 2B). To find genes (cut‐off log fold change of 0.5) that are affected throughout the treatment interval, a Venn diagram was generated overlapping DEGs at D0 to D2 and D0 to D14 (Figure 2C). A total of 160 overlapping genes were found to be regulated at both time intervals.

From those 160 genes, the following genes of special interest based on involvement in T cell pathways or general association with urticaria were identified for further assessment: *CD52*, *COMMD6*, and *CLEC2B* with additionally chosen *IntegrinB3* and *CD28*. The validation of those chosen genes shows a tendency toward upregulation at D2 compared to D0. For each gene, gene expression levels of healthy controls as well as responders (*n* = 14) and nonresponders (*n* = 3) at baseline (D0) were compared (Supporting Information S1: Figure 1). Furthermore, the change in gene expression levels at D2, D14, and D28 compared to D0 for the responder cohort was investigated. Significant regulation was observed in the comparison of healthy controls, responders, and nonresponders where *CLEC2B* is significantly upregulated in responders compared to healthy controls, whereas *CD28* is significantly downregulated in responders compared to healthy controls. For the time course, there is generally a tendency to gene upregulation at D2, but especially *CD52* and *CD28* were significantly upregulated compared to D0.

### 3.4 miRNA Microarray Shows Modest Regulation Changes

**Figure 3:**
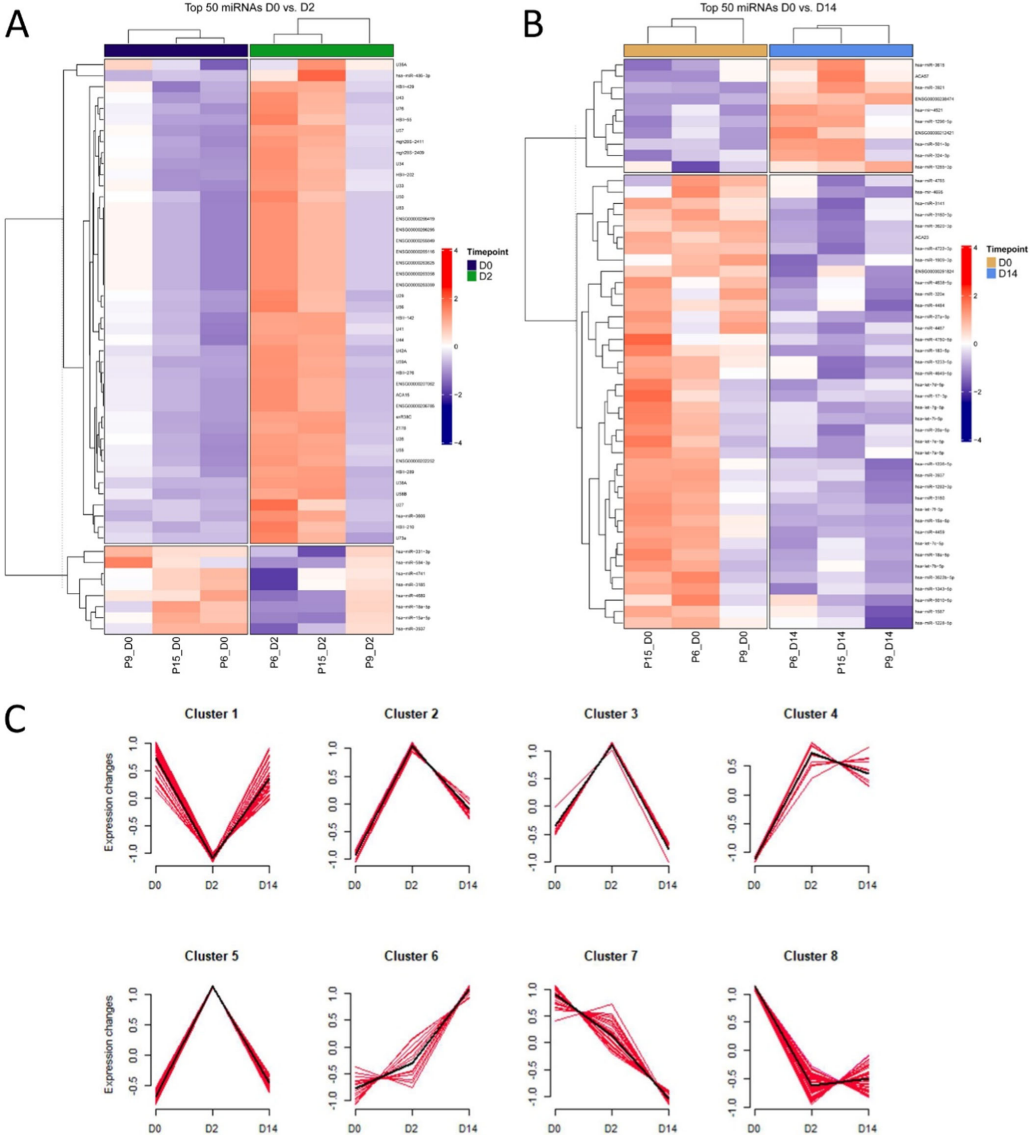
Differential regulation of miRNAs at D2 and D14 compared to D0 after omalizumab treatment. Heat map depicting regulated miRNAs at D2 (A), and D14 (B) compared to D0 based on miRNA microarray analysis. Red color indicates gene upregulation, purple color indicates gene downregulation, and white color indicates regulation in gene expression. Depicted in C is the time series clustering of microRNA expression. Red lines indicate normalized and uniformly scaled values of expression, and black lines indicate the mean trend per timepoint. Membership is defined by a c‐means clustering coefficient > 0.9 for a specific cluster. D = day, P = patient.

Next, the goal was to investigate miRNAs during omalizumab treatment. MiRNA from three responders were investigated at D0 to D2, and to D14, respectively. In comparison to the DNA microarray results, the miRNA results were less prominent, but similarly showed mostly upregulation of miRNA expression. The results were visualized in heatmaps showing the top 50 miRNAs to compare D0 to D2 (Figure 3A), and D0 with D14 (Figure 3B). For the comparison of D0 to D2, and to D14, respectively, the top expressed miRNAs were then assorted into clusters (Figure 3C). For most miRNAs, the strongest changes are seen at D2 after treatment initiation.

**Figure 4:**
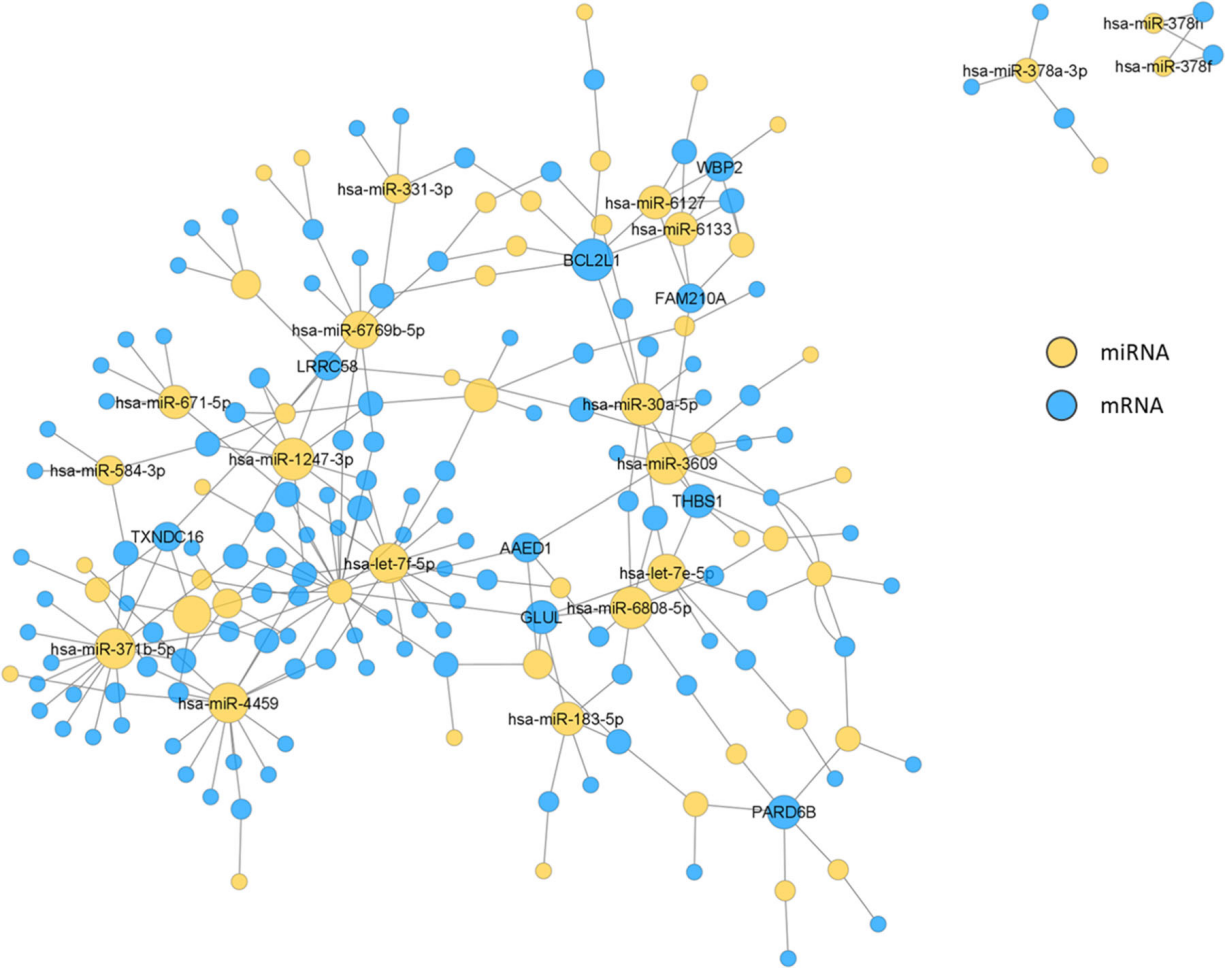
miRNA−mRNA interaction network of significantly regulated genes. Blue nodes: mRNA, yellow nodes: miRNA. Connecting edges indicate predicted miRNA−mRNA interactions based on the Transcriptome Analysis Console software database. Node size is proportional to connectivity. Node labels are reduced for better readability.

Furthermore, data from the DNA microarray analysis (with related mRNA) were correlated with the data from this miRNA microarray analysis. This analysis was illustrated by a co‐expression network (Figure 4), which led to the identification of three miRNAs from two prominent clusters chosen above: hsa‐let‐7e‐5p, hsa‐miR‐3609, and hsa‐miR‐486‐3p. These three miRNAs were investigated on the whole cohort of 17 patients and eight healthy controls. Compared were either healthy controls, responders and nonresponders or the changes in miRNA regulation at D0, D2, D14, and D28 for the responders (Supporting Information S1: Figure 2). The comparison between responders, nonresponders, and healthy controls showed significant differences for hsa‐miR‐3609 between healthy controls and responders and for hsa‐miR‐486‐3p between healthy controls and both responders and nonresponders. There were no significantly altered expressions over the course of the appointed time points observed.

## 4 Discussion

CSU is a common and debilitating disease with a pathogenesis not yet fully understood. Biomarkers are needed to identify suitable current or future treatment options for each patient.

A much‐discussed possible biomarker for CSU is the total serum IgE. Regarding omalizumab treatment, it is thought that by binding to the antibody, the half‐life of IgE is increased and thus, an increase in total serum IgE can be observed, especially in the beginning of the treatment with omalizumab [30]. Altrichter et al. analyzed studies regarding total IgE and CSU and postulated that high IgE levels appear to correspond to long disease duration, high disease activity, and a better response to omalizumab [31]. However, several studies did not share these findings [32, 33, 34]. Here, we detected an overall increase in IgE. We could not observe a correlation with either disease duration, activity, or response to omalizumab.

In this study, we investigated several differentially expressed mRNAs and miRNAs in omalizumab responders first using microarray and then qPCR analyses. Here, we took to look at the changes not only in mRNA but also in miRNA expression at different time points in the first month of treatment with omalizumab additionally comparing responders to nonresponders and healthy controls at baseline. Comparing expression levels at baseline, D2 and D14 by using microarray analysis, it was observed that upregulation outnumbered downregulation and that most upregulation took place on Day 2 under omalizumab treatment. This would correspond to clinically visible amelioration of symptoms as soon as a few days after treatment initiation in some of the patients.

In the first part of our validation study, we investigated changes in gene expression levels based on mRNA microarray results. *CD52* showed to be upregulated at D2—it is expressed on T and B cells as well as, to a lesser extent, on eosinophils and MCs and is known to be responsible for T cell co‐stimulation and migration [35]. A T cell involvement (especially T_H_2) in CSU has been described by several groups [36, 37, 38]. A recent study investigating peripheral blood lymphocytes in CSU patients during omalizumab treatment has found both memory CD4 + T cells as well as effector CD8 + T cells upregulated under treatment [38]. Another recent study has focused on chemokines in skin biopsies of CSU patients and found a strong increase of CD4^+^CCR5^+^ T cells compared to healthy skin [39]. Additionally, MCs carrying the ligand for CCR5, namely CCL3, were also increased in CSU skin and found in close proximity to T cells [39]. Omalizumab itself was reported to influence T cell cytokines such as IL‐31 [40]. Thus, it was interesting whether our data indicates transcriptomic changes on T cell level during the first month of treatment and here especially during the first week. This is why we also investigated *IntegrinB3* and *CD28*. *CD28* was significantly upregulated at D2. It has an important role in signal amplification of T cell stimulation, affects T cell proliferation and differentiation and was already described in association with CSU by Brzoza et al. exploring CSU on a genetic level [41, 42]. Integrin β3, a member of the integrin transmembrane receptor family, was described to play a role in skin inflammation [43, 44]. We observed a significant downregulation of *IntegrinB3* expression levels at D14, which could correlate with reduced inflammation seen under omalizumab therapy.

*COMMD6* was one of the DEGs proposed by our microarray analysis and showed an upregulation tendency at D2 when looking at the cDNA from the whole cohort; the same tendency could be observed with *COMMD8* (data not shown). These genes have not yet been described in association with CSU and could thus be considered a new finding. Both genes code for proteins of the copper metabolism gene MURR1 domain family and act as NF‐κB‐inhibitors [45]. COMMD6 has been described to be linked to the activation of NF‐κB signaling pathways in tumors [46]. COMMD8 has been investigated in venom immunotherapy and grass pollen allergen immunotherapy as a possible inductor of NF‐κB tolerance [47].

A very different direction in omalizumab treatment and, thus, CSU pathogenesis was explored by looking at CLEC2B. It is found on platelets and is thought to be responsible for the preservation of vascular integrity, especially during inflammation by allowing vascular permeability [48]. Wheal development is a central symptom of CSU. Our responders showed significantly increased expression of *CLEC2B* compared to the healthy controls. Interestingly, the previously described Integrin β3 is expressed on platelets, as well.

The second part of our study explored candidate miRNAs from our microarray analysis. miRNAs are thought to be promising biomarkers due to their function as regulators of immunity and inflammation. Only a few studies so far have focused on possible relevant miRNAs for CSU. Interestingly, the top differentially expressed miRNAs identified in our microarray and network analyses have not been linked to CSU so far. Hsa‐let‐7e‐5p was reported to be differentially regulated in oral lichen planus—a disease with pathogenesis not yet fully understood, however, where T cells play a crucial role [49]. Furthermore, de la Rica et al. reported on the cluster of hsa‐let‐7e‐5p and miR‐125a‐5p in the involvement of osteoclast differentiation by regulating NF‐κB [50]. miR‐125a‐5p was observed to be significantly upregulated in CSU by Zhang et al. comparing miRNA microarray data of 20 CSU patients and 20 healthy controls [24]. However, miR‐125a‐5p did not appear to be significantly regulated in our study.

Of the other differently expressed miRNAs in our cohort, little is known so far. Bianchi et al. investigated miR‐486‐3p and found it to promote granulocyte differentiation [51]. Hsa‐miR‐3609 was investigated in different tumor entities [52]. So far there is, to our knowledge, no published research on their possible roles in inflammatory or autoimmune diseases.

The differences in the proposed miRNAs in this miRNA microarray analysis compared to other miRNA candidates are possibly the ethnicity of the cohort (most of the currently published CSU miRNA studies investigated an Asian population cohort), and the differences in the overall pool of miRNAs provided by the respective chip for the microarray analyses. Another crucial difference is the study layout: We investigated patients under omalizumab treatment, which the other studies did not, and examined total miRNAs.

This study has some limitations. The primary limitation is the sample size, particularly regarding the number of nonresponders. However, thus our study presents data from real‐life conditions with, at times, limited patient compliance. Another limitation is the relatively short time frame of this study knowing that there are patients who are late responders to omalizumab—a bigger time frame would certainly provide more information on possible mRNA and miRNA regulations probably leading to a clearer determination of biomarkers in the treatment of CSU.

Nevertheless, to the knowledge of our study group, a study setting containing microarray analysis and validation at different time points of the first month of treatment with omalizumab has not yet been performed and may thus provide new input for further research.

Our study looked beyond the IgE influence of omalizumab on CSU and proposed new evidence of its effect on T cells by identifying and validating several possible mRNA and miRNA candidates. Our results suggest its possible effect on vascular integrity, which has not been thoroughly investigated yet. Our research thus shows new insights on omalizumab treatment and thus might provide potential perspectives in the search for biomarkers for CSU. Further studies investigating larger, ethnically diverse patient cohorts are needed to further deepen our knowledge of the disease.

## Author Contributions

**Anna Smola:** investigation, methodology, formal analysis, data curation, visualization, writing–original draft, writing–review and editing. **Heike C. Hawerkamp:** investigation, methodology, formal analysis, data curation, visualization, supervision, writing–original draft, writing–review and editing. **Péter Oláh:** formal analysis, data curation, software, validation, visualization. **Andreas Kislat:** investigation, methodology, formal analysis, data curation, validation, visualization, writing–original draft. **Nicole Duschner:** investigation, methodology, data curation, validation. **Bernhard Homey:** resources, writing–review and editing. **Stephan Meller:** conceptualization, funding acquisition, resources, supervision, writing–original draft, writing–review and editing.

## Ethics Statement

This study was approved by the local ethics committee of the medical faculty of the Heinrich‐Heine‐University Duesseldorf (No: 4676). All patients provided written informed consent prior to study participation.

## Conflicts of Interest

The authors declare no conflicts of interest.

## Supporting information

Method description for cDNA generation and qPCR, qPCR primers list and details on statistical analysis for qPCR data can be found in the supplement as well as additional qPCR data (Supplemental Figure 1: qPCR analysis of CD52, CD28, COMMD6, CLEC2B and IntegrinB3. Supplemental Figure 2: qPCR analysis of hsa‐let‐7e‐5p, hsa‐miR‐486‐3p and hsa‐miR‐3609). *(Supplementary material)*.

## Data Availability

The data that support the findings of this study are available from the corresponding author upon reasonable request.
