# Supplementary material for: Omalizumab Treated Urticaria Patients Display T Cell and Thrombocyte‐Associated Gene Regulation
Source: Immun Inflamm Dis. 2025 Feb 11;13(2):e70132. doi: 10.1002/iid3.70132 (PMC11813983; doi:10.1002/iid3.70132)
Supplement: Supplementary file 1 — Method description for cDNA generation and qPCR, qPCR primers list and details on statistical analysis for qPCR data can be found in the supplement as well as additional qPCR data (Supplemental Figure 1: qPCR analysis of CD52, CD28, COMMD6, CLEC2B and IntegrinB3. Supplemental Figure 2: qPCR analysis of hsa‐let‐7e‐5p, hsa‐miR‐486‐3p and hsa‐miR‐3609). (Supplementary material). [file IID3-13-e70132-s001.pdf]

Supplemental Information

Omalizumab treated urticaria patients display T cell and thrombocyte-associated gene regulation

Anna Smola, MD[1]*, Heike C. Hawerkamp, PhD[1]*, Péter Oláh, PhD[1,2], Andreas Kislat, PhD[1], Nicole Duschner, MD[1], Bernhard Homey, MD[1], Stephan Meller, MD[1]

[1] Department of Dermatology, Medical Faculty, Heinrich-Heine-University, Duesseldorf, Germany

[2] Department of Dermatology, Venereology and Oncodermatology, University of Pécs, Pécs, Hungary

*Equal contribution

Corresponding author:
Heike C. Hawerkamp
Department of Dermatology, Medical Faculty, Heinrich-Heine-University Duesseldorf
Moorenstr. 5
D-40225 Duesseldorf, Germany
e-mail: heike.hawerkamp@hhu.de

Supplemental Material and methods

CDNA generation and qPCR

For cDNA synthesis of miRNA, the miRCURY® LNA® RT Kit (Qiagen; Venlo, Netherlands) was used according to manufacturer's instructions. The mRNA was reverse transcribed into cDNA as described previously[1], and both cDNAs (from mRNA and miRNA) were analysed via qPCR using a QuantStudio 6 Flex system (Thermo Fisher Scientific)[1].

QPCR primers

The following qPCR primers for mRNA were used: CD28 (forward: 5' gagaagagcaatggaaccattatc 3', reverse: 5' tagcaagccaggactccaccaa 3'), CD52 (forward: 5' cctcttcctcctactcaccatc 3', reverse: 5' ctggtgtcgttttgtcctga 3'), CLEC2B (forward: 5' tggggctttaagagtgaagg 3', reverse: 5' ttgggtaaagccagttagcaa 3'), COMMD6 (forward: 5' ccaagtgctttgaaatgacg 3', reverse: 5' agcagaaaggagactggaggt 3'), COX7B (forward: 5' agcgcactaaatcgtctcca 3', reverse: 5' gaaaatcaggtgtacgtttctgg 3'), IntegrinB3 (forward: 5' ccatgatcggaaggagtttgct 3', reverse: 5' aaggtggatgtggcctctttat ac 3'). Furthermore, the endogenous control 18S rRNA primer-probe set (ThermoFisher Scientific) were used.

For miRNA analysis the following primer from Qiagen were used: UniSp6 (Control; Cat#: YP00203954), hsa-let-7e-5p (YP00205711), hsa-miR-486-3p (YP00204107), and hsa-miR-3609 (YP02107756).

Statistical analysis

Analysis of qPCR data – For qPCR analysis, Mann Whitney *U* test or Kruskal-Wallis test with Dunn's corrections were used to calculate statistical differences. P values ≤ 0.05 were considered significant.

Supplemental Figures

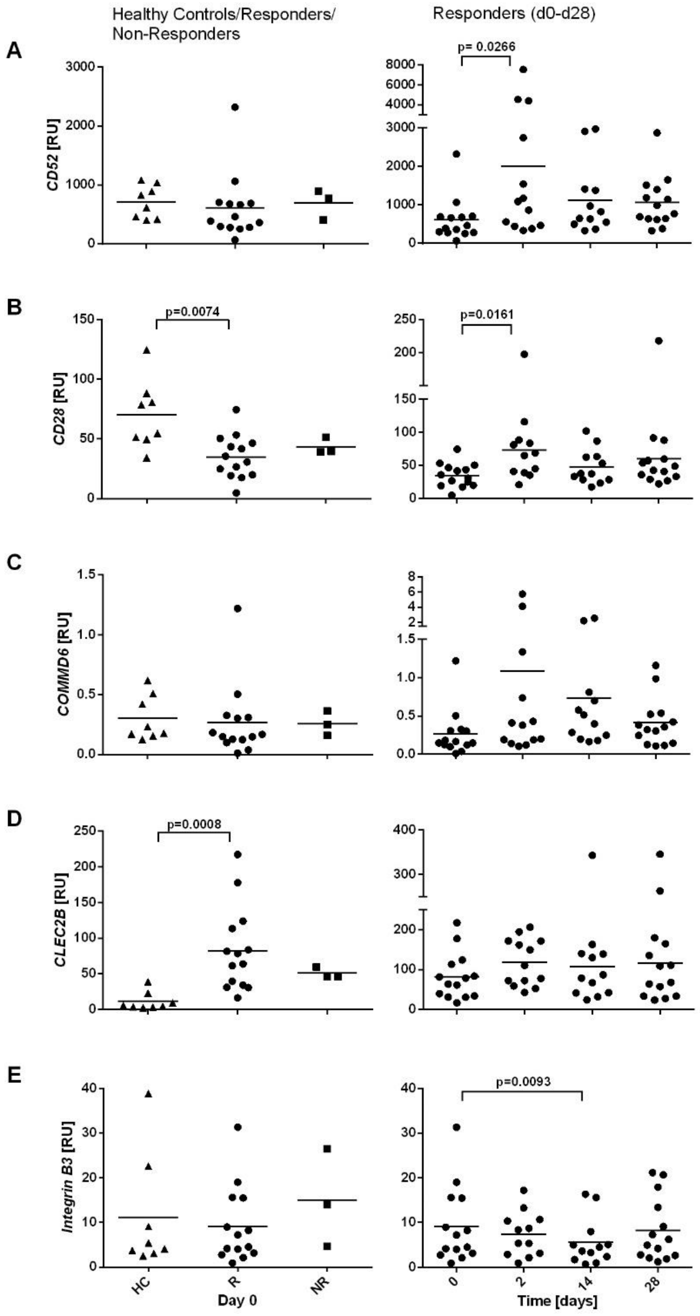

Supplemental Fig. 1**: qPCR analysis of *CD52, CD28, COMMD6, CLEC2B and IntegrinB3.*** Depicted are gene expression levels of *CD52* (A), *CD28* (B), *COMMD6* (C), *CLEC2B* (D) and *IntegrinB3* (E) comparing either R, NR (n=3) and HC (n=8) or the changes of gene expression levels for the respective gene at D0, D2, D14 and D28. Comparing R, NR and HC the Kruskal-Wallis test with Dunn's post-test was used. For the changes of gene expression at D2, D14 and D28, each was compared to D0 using the Wilcoxon test. Significant P values were stated above the respective groups. Patients or healthy donors are displayed with individual symbols (triangle, dots, squares) together with a line representing the mean. Data shown in RU compared to 18S. Abbreviations: D = day, RU = relative expression units, R = responder, NR = non-responder, HC = healthy control.

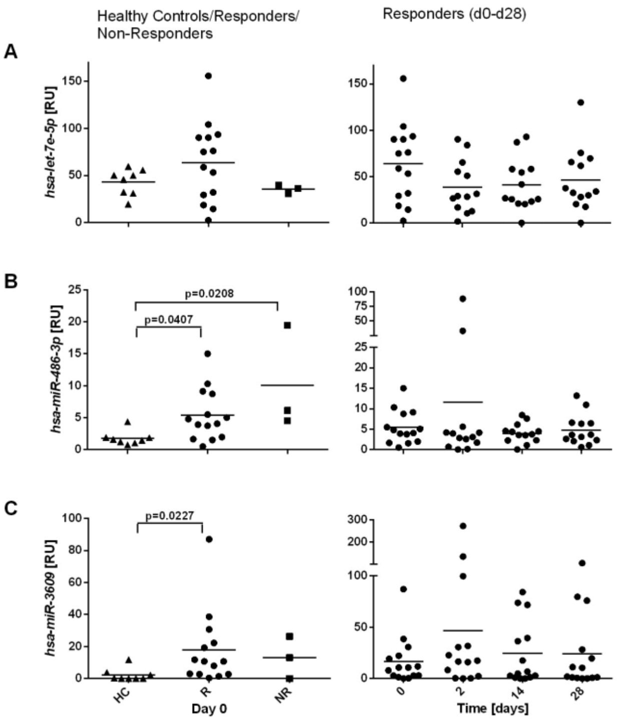

Supplemental Fig. 2**: qPCR analysis of hsa-let-7e-5p, hsa-miR-486-3p and hsa-miR-3609.**
Depicted are miRNA expression levels of hsa-let-7e-5p (A), hsa-miR-486-3p (B), and hsa-miR-3609 (A) comparing either R, NR (n=3) and HC (n=8) or the changes of miRNA expression levels at D0, D2, D14 and D28. Comparing R, NR and HC the Kruskal-Wallis test with Dunn's post-test was used. The changes of miRNA expression at D2, D14 and D28 each compared to D0 were analysed using the Wilcoxon test. Patients or healthy donors are displayed with individual symbols (triangle, dots, squares) together with a line representing the mean. Data shown in RU compared to UniSp6 (control). Abbreviations: D = day, RU = relative expression units, R = responder, NR = non-responder, HC = healthy control.

Supplemental Reference

1 Hawerkamp, H. C. *et al.* Vemurafenib acts as an aryl hydrocarbon receptor antagonist: Implications for inflammatory cutaneous adverse events. *Allergy* **74**, 2437-2448 (2019). https://doi.org:10.1111/all.13972
